# Phosphorus-32, a Clinically Available Drug, Inhibits Cancer Growth by Inducing DNA Double-Strand Breakage

**DOI:** 10.1371/journal.pone.0128152

**Published:** 2015-06-01

**Authors:** Yulan Cheng, Ana P. Kiess, Joseph M. Herman, Martin G. Pomper, Stephen J. Meltzer, John M. Abraham

**Affiliations:** 1 Department of Medicine, The Johns Hopkins University School of Medicine, Baltimore, Maryland, United States of America; 2 Department of Radiation Oncology and Molecular Radiation, The Johns Hopkins University School of Medicine, Baltimore, Maryland; 3 Department of Radiology and Radiological Science, The Johns Hopkins University School of Medicine, Baltimore, Maryland, United States of America; ACTREC, Tata Memorial Centre, INDIA

## Abstract

Radioisotopes that emit electrons (beta particles), such as radioiodine, can effectively kill target cells, including cancer cells. Aqueous ^32^P[PO_4_] is a pure beta-emitter that has been used for several decades to treat non-malignant human myeloproliferative diseases. ^32^P[PO_4_] was directly compared to a more powerful pure beta-emitter, the clinically important ^90^Y isotope. *In vitro*, ^32^P[PO_4_] was more effective at killing cells than was the more powerful isotope ^90^Y (P ≤ 0.001) and also caused substantially more double-stranded DNA breaks than did ^90^Y. *In vivo*, a single low-dose intravenous dose of aqueous elemental ^32^P significantly inhibited tumor growth in the syngeneic murine cancer model (*P* ≤ 0.001). This effect is exerted by direct incorporation into nascent DNA chains, resulting in double-stranded breakage, a unique mechanism not duplicatable by other, more powerful electron-emitting radioisotopes. ^32^P[PO_4_] should be considered for human clinical trials as a potential novel anti-cancer drug.

## Introduction

Beta particles (electrons) emitted by radioisotopes are known to efficiently kill cancer cells. This finding has already been clinically exploited by using ^131^I to treat thyroid cancer [1], a strategy still employed successfully in more than 50% of such patients in the United States, with over a 90% cure rate. Similarly, beta particle-emitting radiolabeled antibodies directed against CD20, including ^131^I-Bexxar (tositumomab) and ^90^Y-Zevalin (ibritumomab tiuxetan), have been used against non-Hodgkin’s lymphoma [2,3]. Moreover, ^90^Y-labeled somatostatin receptor ligand is utilized to treat neuroendocrine tumors [4]. Electrons emitted by ^32^P have an energy level intermediate between those of ^131^I and the more powerful ^90^Y, resulting in a path length of up to 5 mm in human tissues [5]. Electrons emitted from radioisotopes can strike thousands of cells. The resulting bystander effect amplifies the lethal potential of each beta particle emitted in or near a tumor. However, as we shall show below, we have discovered that among all available beta-emitting isotopes, ^32^P possesses a unique chemically and radiologically-based double-strand breakage mechanism, which confers greater anti-tumor efficacy than other beta-emitters of comparable power.

Human cancer-derived cell lines established in immunocompromised mice are a valuable tool for testing the effectiveness of candidate anti-cancer agents [6–9]. We previously found that a single, low-dose intravenous injection of [^32^P]ATP significantly inhibits tumor growth for several weeks in murine xenograft models [10,11]. Because ATP is a small naturally-occurring molecule, its radiolabeled form poses some advantages over larger synthetic compounds as a potential anti-cancer therapeutic, including lower immunogenicity, greater tumor penetration, and superior pharmacokinetics [12]. Inorganic [^32^P]PO_4_, a simple aqueous ion, has been used for decades as a therapeutic agent for polycythemia vera and essential thrombocythemia [13]. This ion was also previously used for palliation of bone pain due to metastases, where it was thought to be incorporated into the extracellular matrix [14]. However, aqueous ^32^P use has never been established as a primary anti-cancer strategy *per se*.

The clinical application of ^32^P was first attempted in the 1930’s [15–18]. Since that time, ^32^P usage has generally been restricted to a colloidal suspension form, wherein ^32^P forms a component of a complex, insoluble particle [19–22]. This form of ^32^P is typically injected directly into the tumor, with the colloidal suspension preventing the radioisotope from leaving the intended target and disseminating throughout the body. The administration of aqueous ^32^P as a primary anti-cancer agent has not been studied, aside from its palliative use for relief of pain due to bone metastases.

Recent experimental findings have led to the development and use of the alpha- and beta-emitter ^223^Ra to selectively target bone metastases in patients with castration-resistant prostate cancer [23,24]. Originally developed by a Norwegian company Algeta, Alpharadin was approved for use in the United States in 2013, and is now marketed by Bayer under the name Xofigo [25]. Thus, ^223^Ra is the latest simple radioactive element to become an effective anti-cancer drug.

We now report that a single, low-dose intravenous injection of aqueous ^32^P results in rapid, significant growth inhibition of pre-established tumor growth in an immunocompetent (syngeneic) murine model. We also show that ^32^P is more efficient than equivalent doses of higher-energy extracellular electrons, such as those emitted by ^90^Y, a beta-emitting radioisotope in common use today. We provide evidence that this higher efficiency results from the direct incorporation of ^32^P into nascent DNA, causing double-strand DNA breakage via a combined chemical-radiological mechanism that cannot duplicated by other beta-emitting radioisotopes, such as ^131^I and ^90^Y. This finding has immediate ramifications for the expanded treatment of primary human cancers.

## Methods

### Measurement of *in vitro* cell killing by ^32^P and ^90^Y

Two thousand cells of the murine BALB/c CRL2836 cell line or the human HeLa S3 cell line were grown in complete medium in a 96-well plate and were exposed at Day 0 to either 0, 1, 2.5, or 5 μCi of ^90^Y radioisotope or the [^32^P]PO_4_ radioisotope in complete medium. After a 24 h incubation, the radioisotope-containing medium was removed and replaced with non-radioactive complete medium (Day 1). WST-1 proliferation assays (Roche Applied Science, Indianapolis, IN) were performed on Days 1, 2, 3, 4, or 5 to directly measure cell growth. Each experiment was performed in triplicate. Cell lines were obtained from the American Type Culture Collection (Manassas, VA) and used within six months of purchase.

### Assessment of double-strand DNA breaks

Ten thousand HeLa S3 cells were seeded onto Lab-TekII chamber slides (Thermo Fisher Scientific Inc., Waltham, MA). At Day 0, cells were treated with 0 or 3 μCi of ^32^P or ^90^Y in complete medium. At Day 1, all wells were gently washed and fresh non-radioactive medium was added. At Day 1, Day 2, or Day 3, cells were fixed with 10% formalin at room temperature for 10 min, washed with PBS for two min, and permeabilized with 0.2% Triton X-100 with 10% FBS in PBS for 15 min. After a rinse with PBS, primary mouse anti-human H2AX antibody at 1:1000 dilution (Millipore, Billerica, MA) was incubated for 1 h at ambient temperature, then washed twice with PBS for 5 min. A dilution of 1:400 goat anti-mouse IgG with Alexa Fluor (Life Technologies, Grand Island, NY) was incubated for 1 h at ambient temperature and washed twice with PBS for 5 min. Cells were stained with Hoechst solution (1:1000).

### Assessement of ^32^P incorporated into DNA

One hundred and fifty thousand mouse CRL2836 or human HeLa S3 cells were seeded onto a six-well cell culture plate and grown for 24 h (defined as Day 0). Cells were then either incubated overnight with ^32^P[PO_4_], grown for 2 d in non-radioactive medium and the DNA extracted (3 Days); or grown for 24 h, incubated with ^32^P[PO_4_] for 24 h, grown for 24 h in non-radioactive medium and the DNA extracted (2 Days); or grown for 48 h, incubated with ^32^P[PO_4_] for 24 hours, washed with complete medium and the nucleic acid extracted (1 Day). DNA was extracted using the DNAeasy Blood and Tissue Kit (Qiagen, Valencia, CA) and aliquots were incubated with or without four units of DNase I (New England Biolabs, Ipswich, MA) for two h at 37°C before the samples were run on a 5% polyacrylamide gel, exposed to film overnight at 4°C and developed.

### Assay for apoptosis in cell lines incubated with ^32^P.

One hundred thousand mouse CRL2836 cells or HeLa S3 cells were seeded into each well of 6-well culture plates and grown for 24 h. Cells were then incubated with 0, 2.5, 5, 10 or 20 uCi ^32^P[PO_4_] in two ml complete medium for 24 h, and non-radioactive medium added for an additional 24 h. Protein was extracted from each well using RIBA buffer (Cell Signaling Technology, Boston, MA), the protein was quantified using a BCA protein assay kit (Pierce, Rockford, IL),and identical quantities were run on a 10 to 20% polyacrylamide gel, and blotted to nitrocellulose. A western blot using a primary antibody to cleaved caspase-3 protein (Cell Signaling Technology, Boston, MA) was used to assay for apoptosis. A second western blot with identical protein quantities was probed with antibody against beta-actin (Cell Signaling Technology, Boston, MA).

### Establishment of mouse tumors

Syngeneic BALB/c mouse tumors were established by injecting 2 X 10^6^ BALB/c tumor CRL2836 cells (American Type Cell Culture, Manassas, VA) in a volume of 0.2 mL (50% Matrigel, 50% 1 X PBS) subcutaneously in the left rear and right rear flank. All mice were female, 10 weeks of age, and purchased from Charles River Laboratories (Wilmington, MA).

### ^32^P-Mediated tumor growth inhibition

After ten days, during which time well-vascularized tumors became well-established, an injection of 5 μCi of the monophosphate form of ^32^P (Perkin-Elmer, Cat. # NEX06000, Waltham, MA) was injected intravenously *via* the tail vein in 0.1 mL of 1 X HBSS. Six tumors (three animals) were studied per each group. After injection, tumor growth was measured three times per week with a digital caliper and the volume was determined using the formula: volume = ½(width)^2^ X (length). Mice were maintained at the Johns Hopkins University Facility in accordance with Laboratory Animal Resources Commission standards under the supervision and approval of the Johns Hopkins University Institutional Animal Care and Use Committee (IACUC).

### Statistical analysis

The data from the WST-1 cell proliferation were presented as means ± standard deviation, and the significance was determined using the unpaired Student’s *t* test. Tumor volumes of the untreated and treated mice were displayed as means ± SE; no outliers were excluded for any reason. Significance was determined using the unpaired Student’s *t* test.

## Results

**Fig 1:**
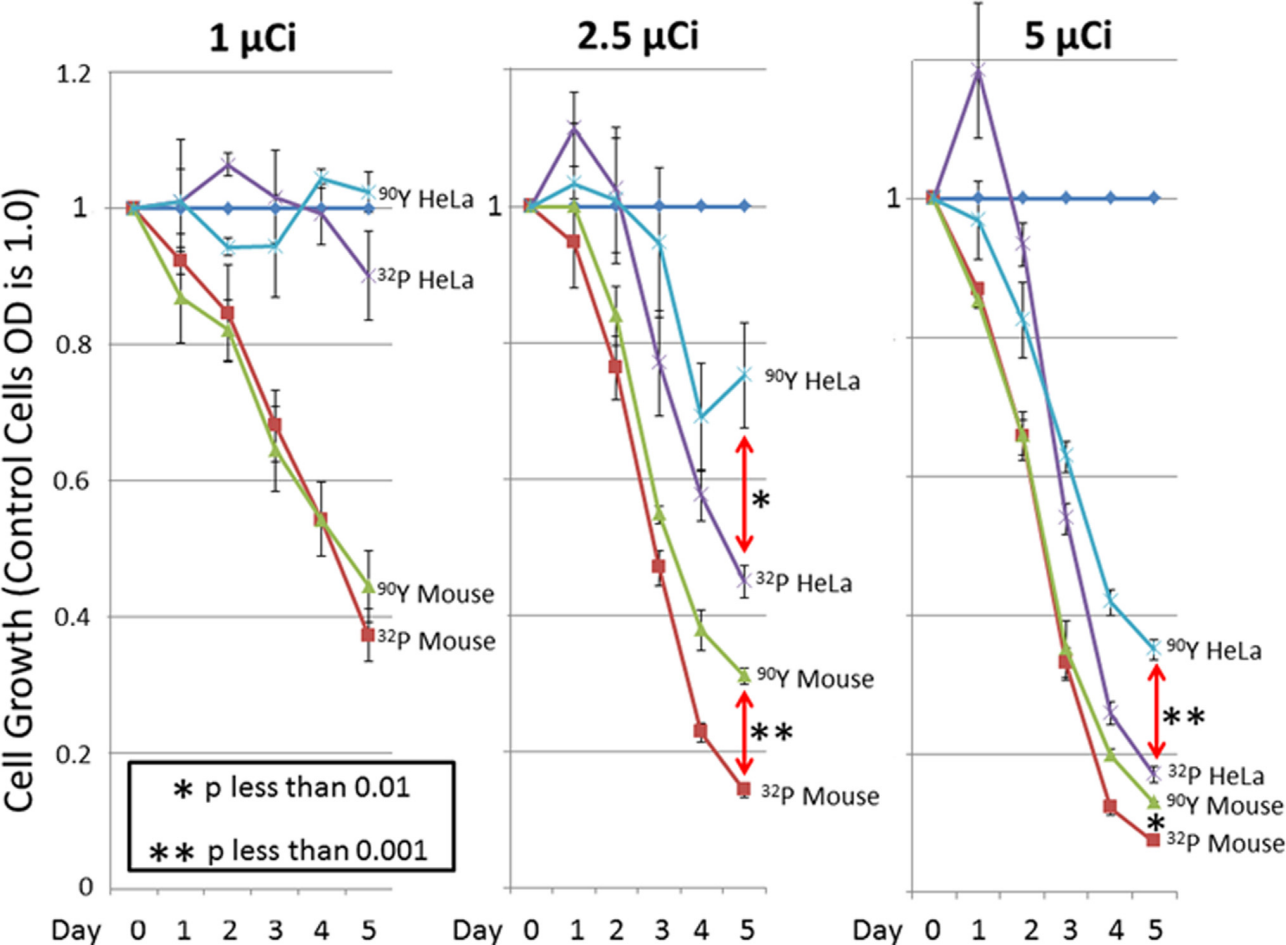
Inhibition of cell growth by [^32^P] PO_4_ or [^90^Y]. The WST-1 proliferation assay was done to determine the level of cell killing by ^32^P or by ^90^Y. BALB/c tumor CRL2836 cells or HeLa S3 cells were exposed to 0 Ci, 1 μCi, 2.5 μCi, or 5 μCi in complete medium. After 24 hours, the medium was changed and cells were grown in non-radioactive complete medium. WST-1 cell proliferation assays were done at Days 1, 2, 3, 4, and 5 in triplicate. The mean is shown plus/minus the standard deviation. The student’s two-sided t-test determined the *P* value shown.

Cells exposed to [^32^P]PO_4_ were compared with those exposed to identical counts per minute of the more powerful beta-particle emitter, ^90^Y, after which WST-1 cell proliferation assays were performed (Fig 1). Different cell lines were expected to demonstrate varying levels of susceptibility to radioisotopes. The 1 μCi dose showed that HeLa cells were less susceptible to beta-emitting isotopes than were BALB/c mouse CRL2836 cells, which originated as an osteosarcoma and were isolated after it had metastasized to lung. Both the 2.5 μCi and 5 μCi doses demonstrated similar results in both cell lines. Although ^90^Y would had been expected to be more lethal than ^32^P (based on its higher-energy electrons), ^32^P killed cells more efficiently than did ^90^Y. In comparisons of the 2.5 μCi and 5 μCi doses, [^32^P]PO_4_ produced survival rates at Day 5 that were barely half of those produced by ^90^Y.

**Fig 2:**
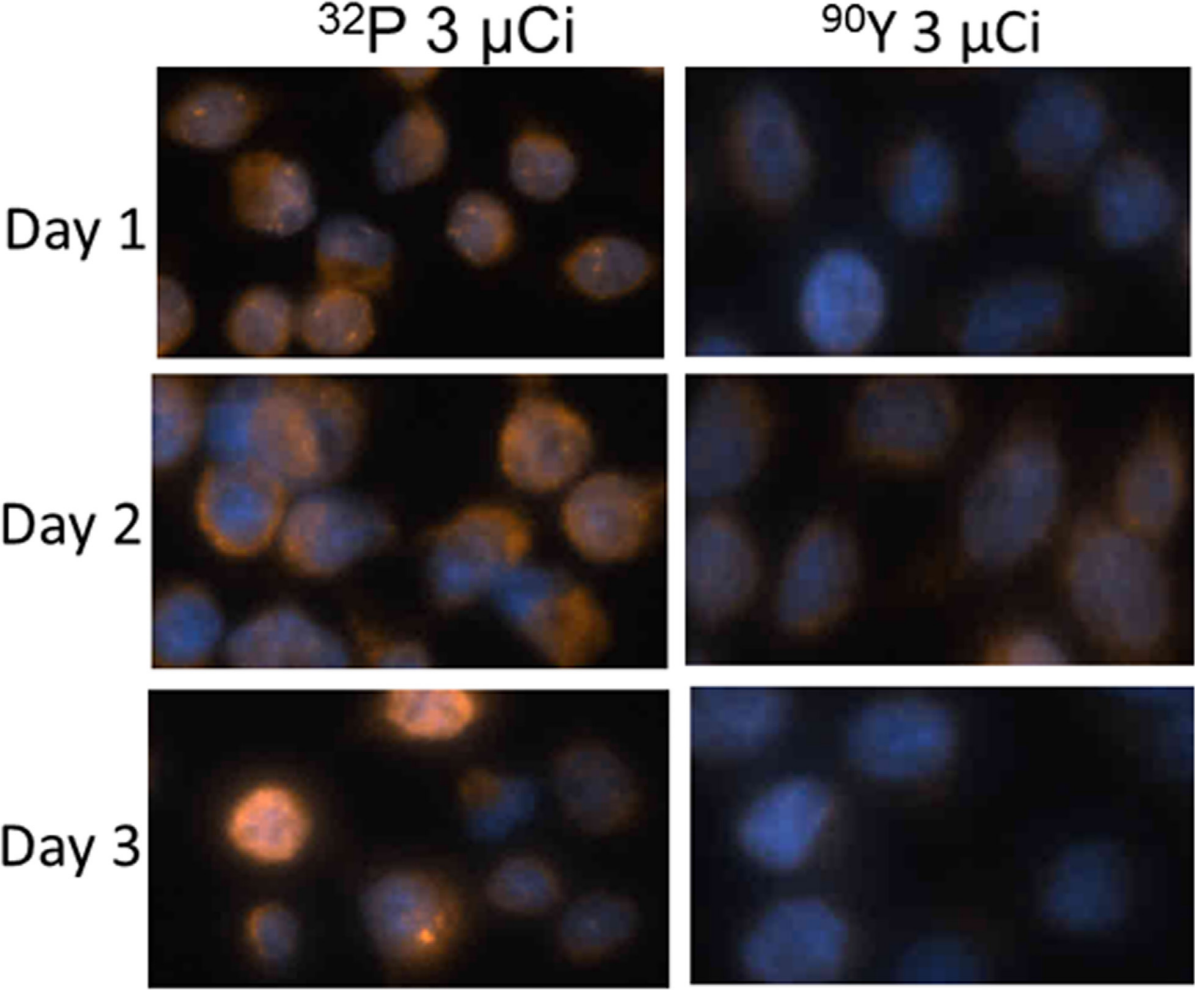
Determination of double-strand DNA breaks in cells caused by exposure to [^32^P]PO_4_ or ^90^Y. HeLa S3 cells or mouse BALB/c CRL2836 cells were grown in multiple sections of chamber slides and exposed to 0 μCi or 3 μCi of [^32^P]PO_4_ or ^90^Y in complete medium at Day 0. After 24 hours, the medium was changed and cells were grown in non-radioactive complete medium. At Day 1, 2, or 3 the presence of double-strand DNA breaks in the cells was determined by staining for phosphorylated H2-AX histones which indicate double-strand DNA damage.

**Fig 3:**
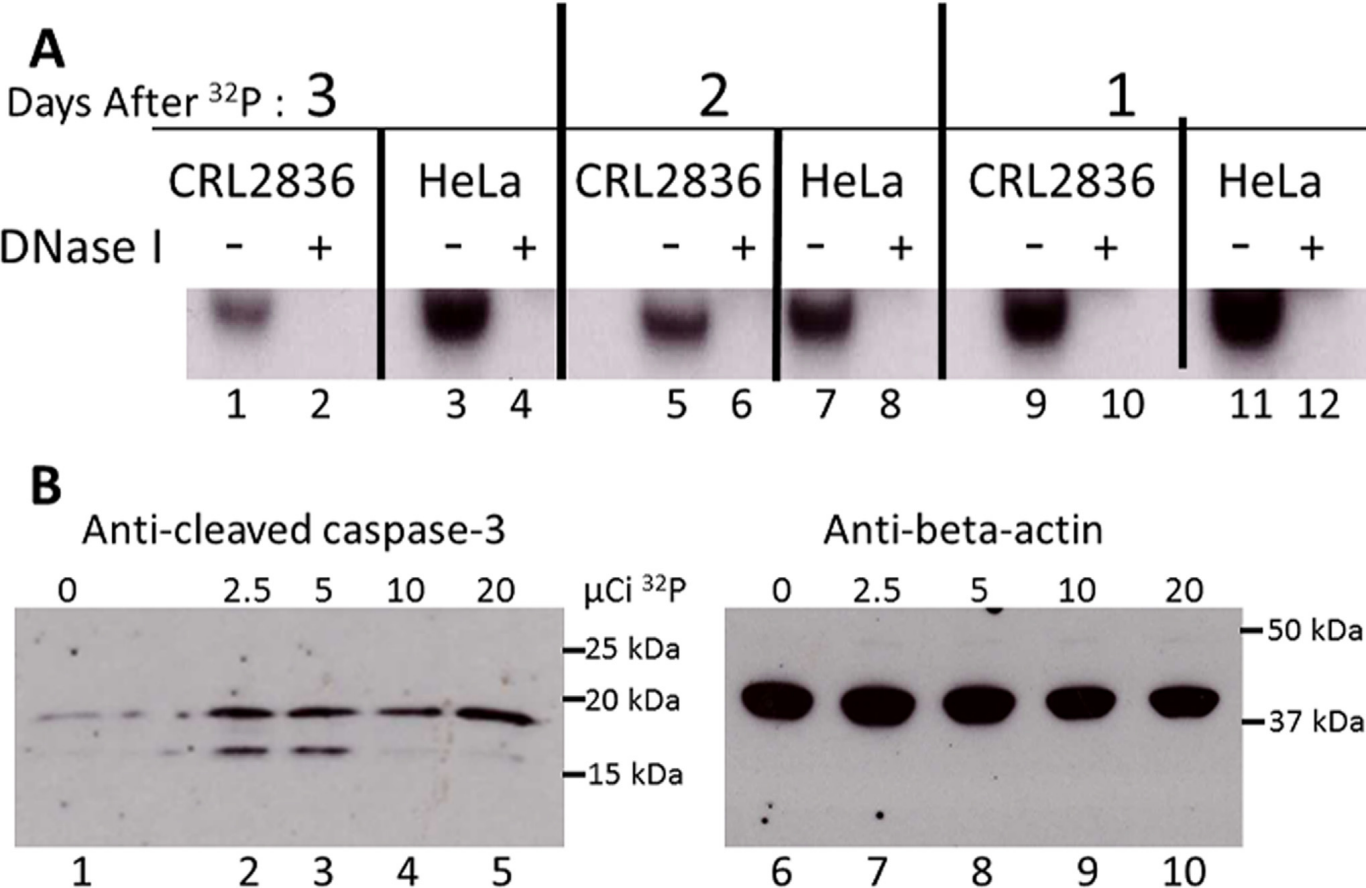
Characterization of ^32^P uptake by the cell. A. ^32^P is directly incorporated into cellular DNA. Mouse CRL2836 or human HeLa S3 cell lines were incubated overnight with ^32^P[PO_4_] and then grown for 48 h in non-radioactive medium (lanes 1 through 4), or grown for 24 h in non-radioactive medium, grown for 24 h with ^32^P[PO_4_], and then grown for 24 h in non-radioactive medium (lanes 5 through 8), or grown for 48 h in non-radioactive medium, then grown for 24 h with ^32^P[PO_4_] (lanes 9 through 12). The extracted nucleic acids were incubated with DNase I, the digestion products were run on a 5% polyacrylamide gel and exposed to film. B. Apoptosis induced by ^32^P in mouse CRL2836 cells. Mouse CRL2836 cells were incubated with 0, 2.5, 5, 10 or 20 μCi ^32^P[PO_4_] for 24 h, and non-radioactive medium added for an additional 24 h. Protein was extracted from each well and analyzed for apoptosis by western blots using antibody to cleaved caspase-3 protein (Lanes 1 through 5). Antibody against beta-actin was used to verify identical amounts of protein were loaded (lanes 6 through 10).

The H2AX assays were used to compare double-strand DNA breakage in cells incubated with ^32^P *vs*. ^90^Y (Fig 2) [26,27]. This assay accurately detects breakage in both strands of DNA at the same genomic locus. Nuclear staining of HeLa S3 cells demonstrated substantial, time-dependent double-strand DNA breakage in cells exposed to ^32^P, while those exposed to identical levels of ^90^Y-based radiation had much less or no detectable DNA double-strand breakage. Digestion with DNase I showed that administered ^32^P had been directly incorporated into cellular DNA (Fig 3A). More than half of the ^32^P retained by the cells that were incubated with ^32^P[PO_4_]for 24 h and then grown in non-radioactive medium for 48 h before the DNA was extracted had been permanently incorporated into cellular DNA. To determine whether cell death involves apoptosis as well as necrosis, mouse CRL2836 cells or HeLa S3 cells were incubated with varying amounts of ^32^P for 24 hours, replaced with non-radioactive medium for an additional 24 hours, and the cells analyzed by western blot for the presence of cleaved caspase-3 indicating apoptosis (Fig 3B). The CRL2836 cells clearly demonstrated that apoptosis was involved in cellular death, while the HeLa S3 cells showed no detectable cleaved caspase-3 (data not shown). Antibody directed against beta-actin was used to verify equal loading of protein amounts in the gel wells. HeLa S3 cells express E6 from HPV18 and are rendered p53 null which severely inhibits apoptosis functions [28,29].

**Fig 4:**
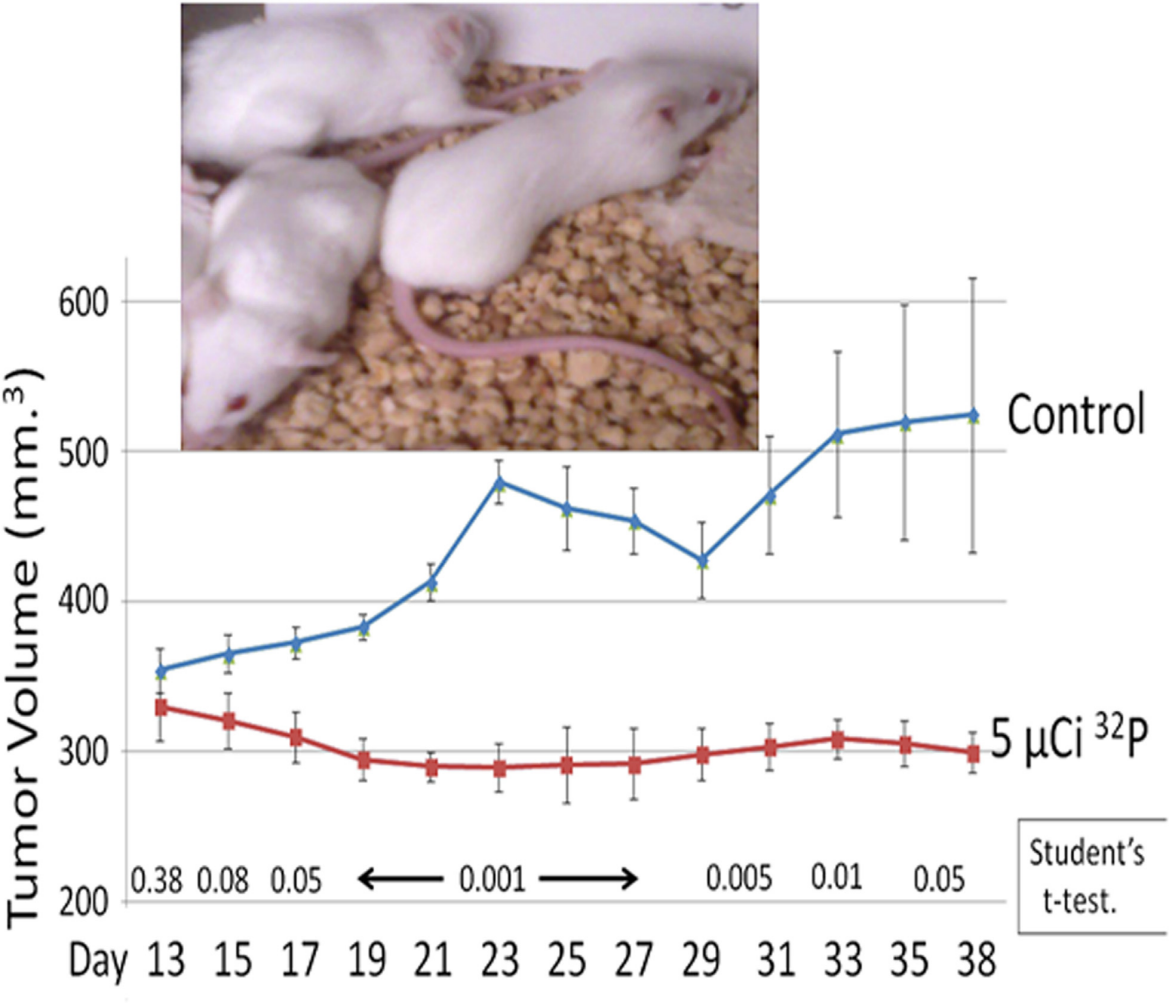
Inhibition of BALB/c syngeneic tumor growth by [^32^P]PO_4_. Syngeneic BALB/c CRL2836 tumors were established in the rear flanks of BALB/c mice at Day 0. After ten days, during which the tumors became well vascularized, mice received an injection of 5 μCi of [^32^P]PO_4_ intravenously via the tail vein. The tumor volumes are shown as the mean of six tumors plus/minus the standard error of the mean. The student’s two-sided t-test determined the *P* value shown. Inset: Representative picture at 35 days post CRL2836 cell injection, showing two control mice on the left, and one mouse that received one 5 uCi [^32^P]PO_4_ dose (right).

Previously, we demonstrated significant inhibition of HeLa S3 cell xenograft growths in nude mice by a single low-dose intravenous (IV) injection of [^32^P]ATP. Here, we chose immunocomponent syngeneic BALB/c mice to more closely recapitulate human malignancy. A single IV injection of aqueous [^32^P]PO_4_ significantly inhibited established syngenic tumor growth in BALB/c mice (Fig 4). There were no apparent detrimental effects of [^32^P]PO_4_ comparing the weight of the treated mice to the control groups (data not shown).

## Discussion

This study documents our discovery that a single intravenous dose of the ^32^P radioisotope significantly inhibits the growth of pre-established tumors in a murine syngeneic model, while simultaneously establishing the mechanism underlying this anti-cancer effect. Specifically, we show that aqueous ^32^P is incorporated into nascent DNA, where isotopic decay shears both strands, causing double-strand breakage as proven by phosphorylation of the histone H2-AX. Our *in vitro* experiments also demonstrate that the pure beta-emitter ^32^P is superior to the more powerful pure beta-emitter ^90^Y in tumor cytotoxicity, and finally, that apoptosis contributes to this cytotoxicity.

**Fig 5:**
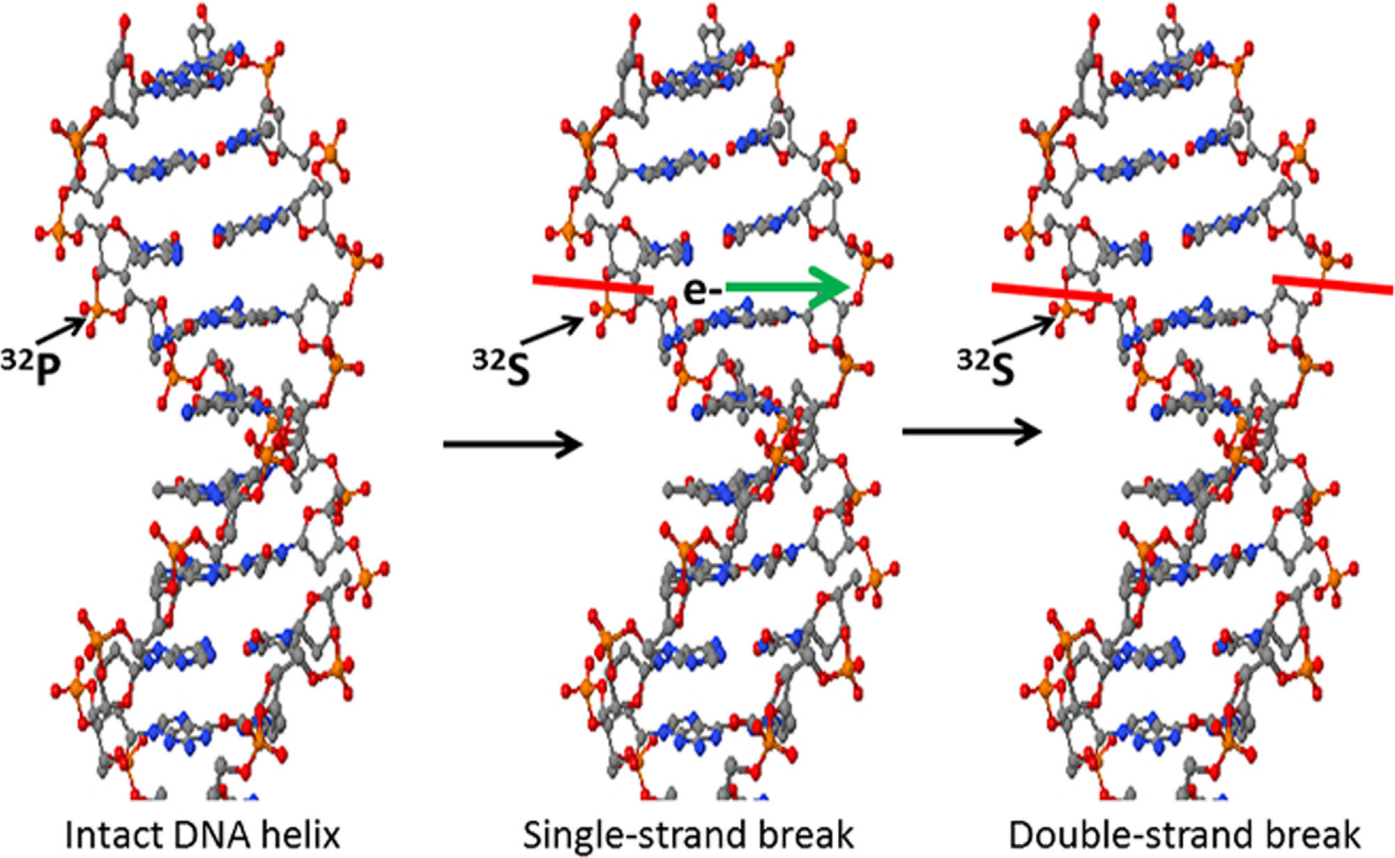
Model of double-strand DNA break after incorporation of [^32^P]PO_4_. The radioisotope is incorporated into the ribose-phosphate backbone of DNA in dividing cells. The process of decaying to sulfur (^32^S) breaks the backbone bond of the initial strand at a 67% rate and releases a high energy beta particle (electron) that must only travel two nm across the helix to the opposite target strand. Although an emitted electron that travels in the perfect orientation from this ^32^P decay to sever the opposite strand will only occur at a low percentage of the time, it is still much higher and more efficient than those electrons which are generated by other beta-producing radioisotopes on the cell surface or in the cytosol that must travel distances that are usually one thousand times or more longer in length.

Fig 5 depicts a proposed mechanism for ^32^P-induced cell killing. In this schematic, ^32^P is incorporated directly into one strand of replicating DNA. Radioactive decay of ^32^P to ^32^S causes chemical breakage of that same DNA strand. Next, the electron released by this decay event needs to travel only 2 nm to reach the contralateral strand of the double helix, severing it and thus causing a double-strand break at this genomic locus. This mechanism stands in stark contrast to non-incorporated beta-emitting radioisotopes, where only a small fraction of emitted electrons travel in the precise orientation necessary to strike one strand plus its opposite strand and cause a double-strand DNA break [30,31]. With ^32^P, the extreme proximity of the contralateral target strand to the decay-produced electron makes this double-strand breakage much more likely to occur [32].

Aqueous [^32^P]PO_4_ offers many potential advantages over other anti-cancer therapeutic agents. Firstly, it allows for rapid systemic distribution and incorporation into primary tumors and ^32^P is preferentially absorbed by rapidly proliferating cells, such as cancer cells. In addition, [^32^P]PO_4_ improves on the previous direct injection of particulate colloidal ^32^P into primary tumors, since aqueous [^32^P]PO_4_ allows for a simple intravenous injection [33–35].

Secondly, ^32^P is already an FDA-approved drug, with a known low toxicity profile [36]. Previous clinical studies of ^32^P-orthophosphate [PO_4_] in aqueous solution for polycythemia vera and essential thrombocythemia have established tolerable dose levels, particularly with respect to myelosuppression [36]. In this context, it is noteworthy that no obvious toxic side effects occurred in any of the model systems we have studied to date. The other concern with its use in these benign hematologic disorders has been an increased incidence of subsequent acute myeloid leukemia (AML) [37,38], but in patients with advanced solid tumors this is less of a concern, since subsequent AML occurs in only 10% *ten years* after ^32^P treatment [39]. Moreover, this new indication and method of use for an existing drug saves considerable time and expense, relative to the investment required for new anticancer agents.

Thirdly, in contrast to other beta-emitting isotopes such as ^131^I and ^90^Y, ^32^P is incorporated directly into nascent DNA [40,41]. Our data suggest that this incorporation dramatically increases the cell-killing efficiency of ^32^P, since the decay of incorporated ^32^P to sulfur chemically breaks the first strand of the DNA and the released electron needs to travel only 2 nm to reach its contralateral DNA strand. Thus, this process efficiently causes double-strand DNA breakage, which is required to overcome innate DNA repair pathways and achieve cell death. In contrast to ^32^P, other electron-emitting isotopes (such as ^131^I and ^90^Y) emit electrons from distances of 1,000 to 5,000 nm away from DNA, some 500- to 2,500-fold farther than the distance of an incorporated ^32^P atom from its sister DNA strand [42–44].

It is intriguing to note that early researchers performing Sanger sequencing with [^32^P]dATP in the 1980’s noted that sequencing products required electrophoresis within two days after the sequencing reaction, otherwise bands seemed to disperse and were difficult to interpret [45]. This same principle may operate with ^32^P as an anti-cancer agent. The decay of ^32^P to sulfur chemically shears the strand of DNA into which it is incorporated. We hypothesize that this event, coupled with the extremely close proximity of the incorporated radioisotope to its sister DNA strand, results in a dramatic increase in cell-killing efficiency *vs*. other beta-particle emitters such as ^131^I and ^90^Y, which are not incorporated into nascent DNA. The resulting implications for the potential clinical treatment of primary human tumors are obvious and far-reaching.
